# Wisconsin’s Environmental Public Health Tracking Network: Information Systems Design for Childhood Cancer Surveillance

**DOI:** 10.1289/ehp.7150

**Published:** 2004-08-03

**Authors:** Lawrence P. Hanrahan, Henry A. Anderson, Brian Busby, Marni Bekkedal, Thomas Sieger, Laura Stephenson, Lynda Knobeloch, Mark Werner, Pamela Imm, Joseph Olson

**Affiliations:** ^1^Division of Public Health, Wisconsin Department of Health and Family Services, Madison, Wisconsin, USA; ^2^E-Commerce, University of Wisconsin Division of Information Technology, Madison, Wisconsin, USA

**Keywords:** childhood cancer, environment, exposures, informatics, information systems, public health, surveillance, tracking

## Abstract

In this article we describe the development of an information system for environmental childhood cancer surveillance. The Wisconsin Cancer Registry annually receives more than 25,000 incident case reports. Approximately 269 cases per year involve children. Over time, there has been considerable community interest in understanding the role the environment plays as a cause of these cancer cases. Wisconsin’s Public Health Information Network (WI-PHIN) is a robust web portal integrating both Health Alert Network and National Electronic Disease Surveillance System components. WI-PHIN is the information technology platform for all public health surveillance programs. Functions include the secure, automated exchange of cancer case data between public health–based and hospital-based cancer registrars; web-based supplemental data entry for environmental exposure confirmation and hypothesis testing; automated data analysis, visualization, and exposure–outcome record linkage; directories of public health and clinical personnel for role-based access control of sensitive surveillance information; public health information dissemination and alerting; and information technology security and critical infrastructure protection. For hypothesis generation, cancer case data are sent electronically to WI-PHIN and populate the integrated data repository. Environmental data are linked and the exposure–disease relationships are explored using statistical tools for ecologic exposure risk assessment. For hypothesis testing, case–control interviews collect exposure histories, including parental employment and residential histories. This information technology approach can thus serve as the basis for building a comprehensive system to assess environmental cancer etiology.

Even though the environment is known to play an important role in human health, no comprehensive, integrated, state or national system exists to track the countless hazards, exposures, and ensuing health effects that could be due to environmental factors [Environmental Public Health Tracking Network (EPHTN) 2004]. For example, when environment is broadly defined to include occupational exposures, environmental pollution, and ionizing and ultraviolet radiation, 9% of cancer deaths have been attributed to known environmental causes (Harvard Report on Cancer Prevention 1996). And yet, what ultimately is known may be extremely limited precisely because a comprehensive, ongoing environmental health tracking system linking hazards and exposures to health effects does not exist.

In response to this challenge, the EPHTN program was established to develop a comprehensive environmental public health surveillance system. The program involves

the ongoing collection, integration, analysis, interpretation, and dissemination of data on environmental hazards; exposures to those hazards; and related health effects. The goal of tracking is to provide information that can be used to plan, apply, and evaluate actions to prevent and control environmentally related diseases. (EPHTN 2004)

Public health depends heavily upon information science. Increasingly, modern public health practice requires advanced, networked, computer-assisted technology to process a wide variety of information assets that monitor disease, analyze and detect risks, provide decision support, alert and communicate with those who need to know, continuously educate and train, support and manage public health response, and measure effectiveness. This recognition has brought about the specification and development of the Public Health Information Network (PHIN) by the Centers for Disease Control and Prevention (CDC) and its public health partners (e.g., state and local public health agencies and professional associations, for example, the Association of State and Territorial Health Officials, Council of State and Territorial Epidemiologists, Association of Public Health Laboratories, National Association of County and City Health Officials).

It has been estimated that environmental pollutants are responsible for a substantial attributable fraction of certain childhood diseases and their associated health care costs (Landrigan et al. 2002). The attendant environmental causes for childhood lead poisoning, asthma, cancer, and developmental disabilities alone may account for as much as $64.8 billion or 2.8% of total U.S. health care costs annually (Landrigan et al. 2002). The environmental attributable fraction for childhood cancer is estimated at 5% (range, 2–10%), and annual health care costs are estimated at $333 million (Landrigan et al. 2002).

Indeed, studies of childhood cancer have discovered a number of biologically plausible environmental associations (Zahm and Devesa 1995), including hazardous air pollutants and leukemia (Reynolds et al. 2003), leukemia and pesticide use (Reynolds et al. 2002), leukemia and electric and magnetic fields (Brain et al. 2003), leukemia and ionizing radiation (Axelson et al. 2002), nervous system cancers and parental pesticide exposures (Feychting et al. 2001), and road traffic (benzene exposures) and leukemia (Crosignani et al. 2004).

In Wisconsin an estimated 25,800 new cases of cancer are expected to have occurred in 2003 (American Cancer Society 2004). Approximately 269 will occur in children. Presently, the environmental contribution and etiology of these cases are unknown. Over the years, many of these childhood cancer cases have been the source of numerous requests for labor-intensive, systematic environmental cancer cluster investigations and assessments (Fiore et al. 1990). However, because of the many intrinsic limitations of the “self-selected” cluster analytic approach (Rothman 1990), our experience has resulted in little, if any, insight into the potential causes, environmental or otherwise.

The maturation of networked information systems holds the promise of automating much of public health practice (Yasnoff 2001). By automating practice with advanced information technology, comprehensive surveillance and tracking systems may be created that have the statistical power and considerable information depth needed to understand the operation of complex disease causal factors. To this end, an information technology platform is described that is in development to support environmental public health tracking in Wisconsin. One application of effort is illustrated using cancer registry data.

## Materials and Methods

### Information technology development.

A number of stakeholder committees were established to guide Wisconsin’s PHIN (WI-PHIN) development and establish functional system requirements. Members included public health staff from the Wisconsin Department of Health and Family Services, the University of Wisconsin [university information technology staff (UW-DoIT)], WiscNet (networking provider), University of Wisconsin Medical School, and Wisconsin State Laboratory of Hygiene); other state agencies; local public health services; hospitals, the health insurance industry; and the Wisconsin business community.

The “Wisconsin Idea,” used to develop the state’s PHIN, involves rapid, state-of-the-art technology transfer from the university to government, businesses, and all citizens of the state and nation (Stark 1995). The WI-PHIN program is a 21st century embodiment of the idea, providing cutting-edge information technology services through research and development. UW-DoIT provides the information systems research, development, technical support, and hosting for WI-PHIN.

Wisconsin has combined its financial support from several categorical funding sources to develop a secure, web-based WI-PHIN portal to respond to bioterrorism and all other public health threats. Funding resources have included bioterrorism public health preparedness funding, National Electronic Disease Surveillance System (NEDSS)–NEDSS Base System (NBS) deployment, Wisconsin Maternal and Child Health Program, audiometric newborn screening, environmental public health tracking, among others.

Nationally, the PHIN has nine architectural functional specifications to guide development. NEDSS (2001) also specifies standards for database structure and electronic surveillance systems. Using the PHIN specifications as a guide (CDC 2002), EPHTN program area module (PAM) architectural requirements were developed for the childhood cancer tracking system. Together, the following attributes were applied to the design of the WI-PHIN portal: The EPHTN PAM must include the secure, automated exchange of cancer case data between public health–based and hospital-based cancer registrars; web-based supplemental data entry for environmental exposure confirmation and hypothesis testing; automated data analysis, visualization, and exposure–outcome record linkage; directories of public health and clinical personnel for role-based access control (RBAC) to sensitive surveillance information; public health information dissemination and alerting; and information technology security.

### Surveillance example: hypothesis generation.

Available data systems were inventoried and included systems describing hazards, exposures, health outcomes, and populations at risk. Sources of data included statewide health and environmental monitoring information and nationally available environmental and demographic data sets. Each system was qualitatively evaluated for its ability to be linked with other systems and for its coverage (years, geographic completeness, etc.). These systems would be contained in an integrated data repository (IDR) and linked through common attributes such as time and geographic location. System specifications required the support of two surveillance tracks—hypothesis generation and hypothesis testing—to provide a more complete view of environmental disease risk. Under the first track (hypothesis generation), cancer case data are sent electronically to WI-PHIN to populate the IDR, and basic surveillance/descriptive analyses are performed. Environmental data are then linked, and exposure–disease relationships are modeled using statistical and geographic information system (GIS) tools for ecologic exposure risk assessment. In the second track (hypothesis testing), case follow-back interviews are conducted using secure web-based data entry forms to obtain person-level exposure histories, including parental employment and residential histories, on cases and controls.

Childhood cancer data were obtained from the Wisconsin Cancer Registry for 1990 through 2000 (the most recent available year) (Wisconsin Department of Health and Family Services 2004). Cases were selected where individuals were younger than 20 years of age at diagnosis. Case frequencies were arrayed by cause, and age-adjusted rates were plotted by county for the most frequent cancer types.

Known exposure–disease relationships were ascertained by performing searches of the National Library of Medicine’s PubMed (2004) database along with Internet searches. A work group was established to review findings and determine the web-based interview structure that would obtain risk-confirming and hypothesis-testing person-level exposure data on cases and controls.

To begin work on hypothesis generation, initial ecologic risk assessments were performed by correlating county air pollution exposure estimates (Technology Transfer Network 2002) with county age-adjusted cancer rates. Age-adjusted rates were constructed using the direct method and the 2000 census standard million population (U.S. Census Bureau 2004). A nonparametric correlation was then calculated between the ranks of county air pollutants and the rank of age-adjusted cancer rate.

## Results

### Information technology.

**Figure 1:**
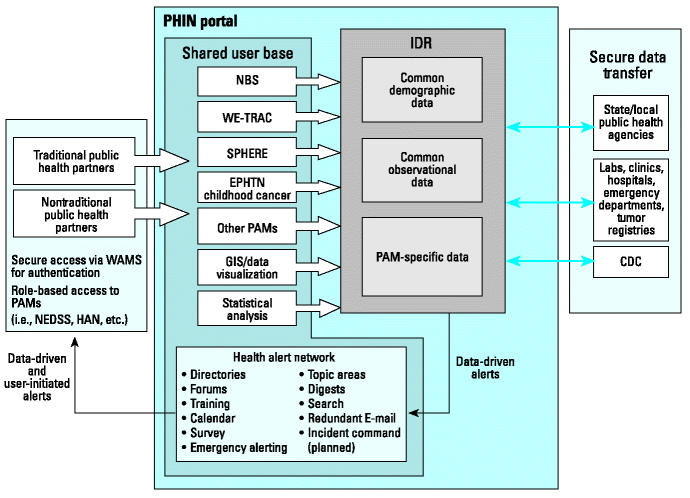
WI-PHIN information flows and services. Abbreviations: HAN, Health Alert Network; PAMs, program area modules; WAMS, State of Wisconsin Web Access Management System.

Substantial progress has been made on the secure WI-PHIN portal since its start in 1999. Since its inception, > $7 million has been expended by combining public health information technology funding sources. Table 1 illustrates the information technology function requirements and Wisconsin’s progress toward achieving them. Figure 1 provides a conceptual diagram of portal information flows and services.

The WI-PHIN has the capability to perform automated data exchange, use electronic clinical data for event detection, and use the web for secure data entry for case follow-up. An online survey capability was created that can support web-based manual entry for case reporting. Storage capacity for laboratory results is established, as is case management capability. Specific case management rules continue to be refined with the integration of PAM business requirements. SAS (version 8.2; SAS, Inc., Cary, NC) has been integrated into the portal for automating statistical analyses and visualization, and GIS services (using Environmental Systems Research Institute, Inc., Redlands, CA, products) continue to be developed. A directory of personnel has been established containing more than 2,400 registered WI-PHIN users from more than 900 agencies (state and local public health agencies, hospitals, local emergency response agencies, clinics, etc.). The directory contains user contact information (e.g., E-mail, phone, fax, pager, cell phone) along with other attributes (public health role, occupation, agency affiliation, professional skills/competencies/certifications, volunteer for emergency response, etc.). Users can create personal groups from the directory and synchronize entries to their personal digital assistants (PDAs).

A considerable alerting and information dissemination capability has been developed. A commercial call-tree service (simultaneous phone, fax, pager, E-mail for public health emergencies and other alerts) has been integrated into the portal. Scenarios are being developed that contact appropriate responders to specific public health emergencies. In addition, the web portal has public and private topic areas and threaded discussion forums that are associated with public health programs such as environmental tracking. Users may bookmark topic areas and receive E-mail updates (digests) when new content is added to their subscribed content areas. All users can easily add content to the portal (text, web links, upload documents, streaming media) and add events to the calendar. Calendar entries can be synchronized to a PDA. Distance training and streaming media services are available within the portal. These features have been used to create on-line courses to train public health staff throughout the state on public health topics (e.g., bioterrorism) and on portal features and use techniques.

Advanced security controls are a part of the portal design. Users must register with the State of Wisconsin Web Access Management System and obtain a login ID and password to access the system. Users then reach the web site with an encrypted secure socket layer (SSL) connection. RBAC determines end-user access to surveillance programs such as the NBS (infectious disease reporting), SPHERE (Secure Public Health Electronic Record of the Wisconsin Maternal and Child Health Program), WE-TRAC (Wisconsin Early Hearing Detection and Intervention Referral and Coordination System), and the EPHTN childhood cancer pilot.

Hardware is also protected. Servers have redundant firewalls, virus scanning, continuous external port scanning and probing, and intrusion detection appliances. The system is continually backed up, and continuity of operations is assured through site mirroring planning and procedural implementation. Administrative security policies cover appropriate conduct and use documents, access auditing and logging, and on-line training.

### Hypothesis generation example: benzene and leukemia.

Table 2 displays the environmental, population, and health outcome data systems that are under evaluation for inclusion and linkage in the EPHTN IDR. Wisconsin childhood cancer cases are displayed in Table 3. A total of 2,960 cases were selected. Leukemia, lymphatic, and brain cancers accounted for 51% of the cases. These were selected for rate analysis, plotting, literature review, and follow-back to assess environmental contributions. More than 1,000 articles/sources were obtained, and environmental exposure history development continues (Agency for Toxic Substances and Disease Registry 2004) for web-based data entry.

**Figure 2:**
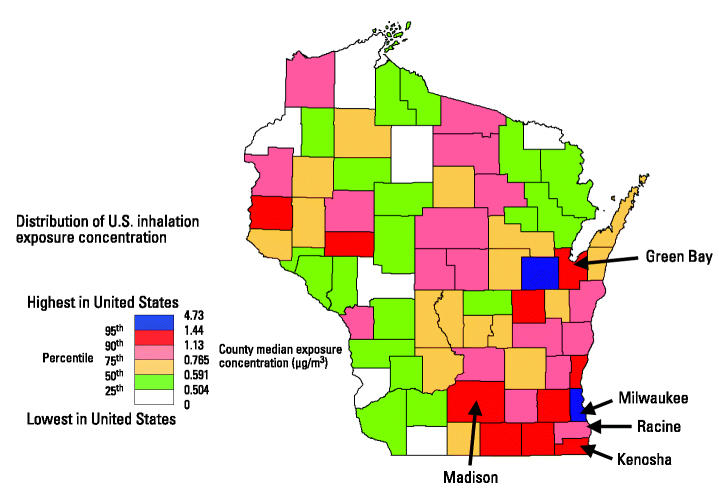
National Air Toxics Assessment: 1996 estimated Wisconsin county median exposure concentration of benzene. From U.S. Environmental Protection Agency/Office of Air Quality Planning and Standards National-Scale Air Toxics Assessment (Technology Transfer Network 2002).

**Figure 3:**
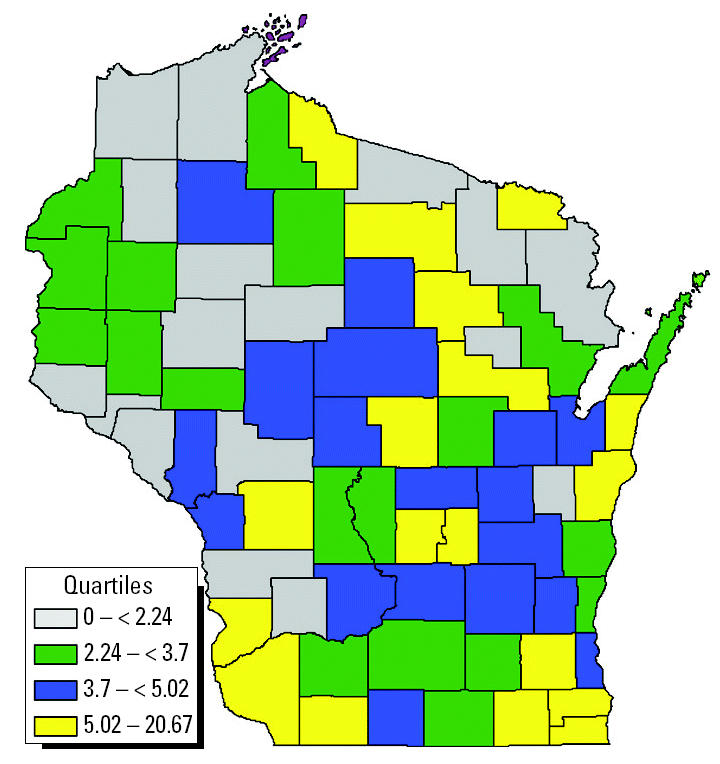
Wisconsin age-adjusted childhood leukemia incidence rates per 100,000 by county, 1990–2000.

A preliminary hypothesis-generating assessment was made with some of the currently available data. These data consisted of the Wisconsin Cancer Registry (Wisconsin Department of Health and Family Services 2004), National Air Toxics Assessment data for 1996 (Technology Transfer Network 2002), and census county population estimates. Age-adjusted county cancer rates were correlated to each of the pollutants. Estimated inhalation concentrations for benzene are depicted by county in Figure 2. Figure 3 plots the age-adjusted leukemia incidence by county. Correlating the two revealed a significant rank correlation between exposure and disease (*R* = 0.31, *p* < 0.01). Indeed, benzene appears repeatedly in the literature as a potential cause of leukemia.

## Discussion

The PHIN information technology functions provide a clear implementation plan to automate public health practice. Automation will be a tremendous benefit to the public health system, improving efficiency, coordination, assurance, response, and evaluation. However, substantial resources are necessary to accomplish this. An EPHTN program meeting the PHIN requirements is only possible by combining multiple funding sources that support public health information technology. Wisconsin has done this and has made substantial progress in its development of WI-PHIN and EPHTN, but much work remains. Continued federal funding and support from other sources will be necessary if the many ambitious PHIN goals are to be achieved.

Wisconsin’s EPHTN childhood cancer pilot PAM has established a PHIN-compatible framework to track the environmental causes of disease. Two mechanisms are specified—hypothesis generating and hypothesis testing. Hypothesis generation occurs through the linking of environmental exposure databases (by time and place) with health outcome and population data. Ecologic risks can be generated that suggest avenues for further investigation. When a subset of the available data was used, a population-level rank correlation was found between estimated human inhalation benzene exposures and childhood leukemia risk, corroborating previous findings. However, the “ecologic fallacy” is the chief limitation of this approach: There can be underlying heterogeneity of exposure levels and covariates within the group or area assigned its population-level fixed value (Rothman and Greenland 1998). Hypothesis testing provides a solution to this by creating a web-based follow-back form to capture individual, person-level exposure histories on cases and controls. Known risks can be assessed on the questionnaire, and hypothesized causes can be tested in the case–control framework. These results can then be used to construct environmental attributable fractions for case incidence.

But both of these approaches are limited in that neither obtains biologic or environmental markers of actual exposures or individual susceptibility. In addition, pre-existing exposure monitoring data may be further limited because much of the available information is collected for regulatory purposes. These environmental monitoring systems have not been designed to substantially support environmental health tracking systems. Reliable and valid laboratory measures of environmental exposures, cancer risk, and individual susceptibility (i.e., gene–environment interactions) are needed, and they would considerably increase our understanding of the environment’s contribution to childhood cancer (Grufferman 1998). Although this detailed environmental monitoring activity is outside of the project scope largely because of funding, the EPHTN PAM is positioned to integrate these kinds of measures because it can accept laboratory result messaging.

Through the Wisconsin Idea, the WI-PHIN program has developed innovative information technology solutions that can serve as an implementation model for others. Best practices and lessons learned are emerging as the WI-PHIN develops its pilot program for environmental childhood cancer tracking. This experience will be shared with other states seeking to better understand the relationship between childhood cancer and the environment using advanced information technology. This approach can then serve as the foundation building toward a comprehensive system to assess environmental cancer etiology while extending the method to tracking other environmental exposure and disease relationships.

## Figures and Tables

**Table 1:** PHIN information technology functional standards and Wisconsin implementation status.

PHIN IT function specification	PHIN standard implementation*^a^*	WI-PHIN status
1. Automated data exchange	Establish ebXML-compliant SOAP web service via an HTTPS connection after appropriate authentication;encrypted messages use industry standard ebXML format and include standardized HL7, version 2.3; HL7, version 3.0; X12; and LDIF message content.	Web service capability established; test deployment with several laboratories.
2. Electronic clinical data: event detection	Data received via ebXML messaging identified in function 1 above stored using NEDSS logical data model specification of the HL7 Reference Information Model and extensions. This allows standards-based interaction with commercial products for reporting, statistical analysis, geographic mapping, and automated outbreak detection algorithms, as well as the processing of queued data from and for electronic messages; the data repository should implement common database technology (e.g., Sybase, Oracle, or SQL Server) running on servers using Windows NT/2000/XP, LINUX, or UNIX and supporting ODBC, ANSI standard SQL, and JDBC access.	Data repository established using Oracle 9i; messaging of laboratory data in pilot/production; hospital tumor registries contacted for case messaging; pilot volunteers identified.
3. Web: manual data entry	Secure browser-based data entry for data input and results other reporting from and to primary care clinical care sites and sources; develop web browser–based data systems using open-platform web servers supporting generic web browsers (HTML 3.0+/Java)	Function established; system operates on Sun Solaris using Weblogic application server; capability will be used to obtain supplemental risk and exposure history data.
4. Laboratory result information	Data stored in HL7-compatible data formats; coding of request and results messages with the LOINC and SNOMED vocabularies; information messaging using function 1.	Storage capability established; vocabulary capability in development.
5. Case management	Using functions 1–4 above, cases should be “linked” and traceable from detection via electronic sources of clinical data or manual entry of case data, and through confirmation via laboratory result reporting.	Capability established; PAM-specific business rules in development for linkage and tracking.
6. Analysis and visualization	Commercial reporting systems integrated using ODBC and JDBC data access; security and access control applied for remote access using SSL and certificate- or token-based authentication with appropriate authentication and authorization.	SAS product integrated; ESRI GIS capability in development; SSL and RBAC established.
7. Personnel directories	Directories present an LDAP version 3.0 standard-based service allowing data access and sharing across multiple computer systems and appropriate organizational boundaries; directory information transfer and sharing supports standard message format (LDIF); data fields use X.500 standards for field type and length.	Capability established; directory contains contact information and roles of > 2,400 registered PHIN users from > 900 organizations.
8. Information dissemination and alerting	Receive, manage, and disseminate alerts, protocols, procedures, and other information for dissemination to public health workers, primary care physicians, public health laboratories, and other partners; ability to “push” information via messages and allow participants to “pull” information via the browsing of secure web sites; support of interactive communication sites for threaded discussion capabilities.	Capability established; call-tree alerting system integrated (voice technologies); public and private topic areas, threaded discussion forums established; push digest subscriptions available from bookmarked topic areas, directing appropriate content to audience.
9. IT Security	Meet/exceed HIPAA requirements; client and server X.509 digital certificates or comparable strong authentication methodology for access; establish RBAC protocols and effective administrative policies; employ desktop/server virus scanning, intrusion detection, network vulnerability analysis, security policy monitoring, regular penetration testing, and active threat intelligence; ensure continuity of operations through planning and procedure implementation.	Capability established, including RBAC, administrative policies, auditing, and training; ongoing virus scanning, intrusion detection, threat intelligence, continuity of operations; independent validation and verification in development; client digital certificates in exploratory phase.

Abbreviations: ANSI, American National Standards Institute; ebXML, Electronic Business using eXtensible Markup Language; ESRI, Environmental Systems Research Institute; HIPAA, Health Insurance Portability and Accountability Act (1996); HL7, Health Level 7; HTTP, Hypertext Transfer Protocol; IT, information technology; JDBC, JAVA Database Connectivity; LDAP, Lightweight Directory Access Protocol; LDIF, Lightweight Data Interchange Format; LOINC, Logical Observation Identifiers; ODBC, Open Database Connectivity; SNOMED, Systemized Nomenclature of Medicine; SOAP, Simple Object Access Protocol; SQL, Structured Query Language.

a From CDC (2002).

**Table 2:** Wisconsin EPHTN data inventory.

Abbreviation	Data set	Scope	Description
AEI	Air Emissions Inventory	State	Emissions from mobile sources
BRRTS	Bureau of Remediation and Redevelopment Tracking System	State	Database of environmental contamination sites including spills, leaking underground storage tanks, state-response sites, and federal Superfund sites
Census	Census	National	Decennial population counts, age, gender, race, census tract, county, ZIP code
DWS	Drinking Water System	State	Drinking-water quality in Wisconsin public wells
GEMS	Groundwater Environmental Monitoring System	State	Environmental monitoring data for Wisconsin landfills, including landfill gas, groundwater, and other sample types
GLAT	Great Lakes Air Toxic Emissions Inventory	Regional	Airborne toxic pollutant emissions affecting air and water quality in eight Great Lakes states
GRN	Groundwater Retrieval Network	State	Groundwater quality in Wisconsin private, public, and monitoring wells
NATA	National Air Toxics Assessment	National	Estimates of 33 air toxics (a subset of 32 air toxics on the Clean Air Act’s list of 188 air toxics, plus diesel particulate matter) (U.S. EPA 1993)
NEI	National Emissions Inventory	National	Hazardous and criteria air pollutants
PEI	Periodic Emissions Inventory	State	Annual emissions of criterion air pollutants and some noncriterion pollutants
RR GIS Registry	Remediation and Redevelopment GIS Registry	State	Sites closed with residual water or soil contamination
SHWIMS	Solid and Hazardous Waste Information Management System	State	Sitings for waste management facilities
SWAP	Source Water Assessment Plan Database	State	Assessment of possible contamination sources within a specified distance from a drinking water well
TRI	Toxics Release Inventory	National	Toxic chemical releases and other waste management activities for specific industry groups and federal facilities
WCR	Wisconsin Cancer Registry	State	Cancer incidence by age, gender, race, county, ZIP code, histology, cytology, staging
WI Hosp	Wisconsin Hospital Discharge	State	Hospitalizations by age, gender, race, county, ZIP code, cause
WMOR	Wisconsin Mortality	State	Mortality by age, gender, race, county, cause

**Table 3:** Wisconsin Cancer Registry 1990–2000: childhood cancer cases frequency by cause (children < 20 years of age).

Cause	Frequency (%)
Leukemia	672 (22.7)
Lymphatic cancers	428 (14.5)
Brain cancer	413 (14.0)
Cervical cancer	283 (9.6)
Bone cancer	174 (5.9)
Soft tissue cancer	151 (5.1)
Kidney and other urinary cancer	126 (4.3)
Thyroid cancer	91 (3.1)
Skin cancer/melanoma and other reportable	88 (3.0)
Other endocrine gland cancer	74 (2.5)
Testicular cancer	66 (2.2)
Eye cancer	59 (2.0)
Ovarian cancer	58 (2.0)
Other central nervous system cancer	47 (1.6)
All other cancers/unknown cancers	42 (1.4)
Oral cancer	33 (1.1)
Peritoneal cancer	27 (0.9)
Liver cancer	24 (0.8)
Nasal cancer	15 (0.5)
Colorectal cancer	14 (0.5)
Other respiratory/thoracic cancer	14 (0.5)
Bladder cancer	13 (0.4)
Bronchus and lung cancer	11 (0.4)
Other female genital cancer	11 (0.4)
Prostate cancer	7 (0.2)
Small intestine cancer	3 (0.1)
Breast cancer	3 (0.1)
Uterine cancer	3 (0.1)
Other leukemias	3 (0.1)
Stomach cancer	2 (0.1)
Pancreatic cancer	2 (0.1)
Laryngeal cancer	1 (0.0)
Pleural cancer	1 (0.0)
Other male genital cancer	1 (0.0)
Total	2,960 (100.0)
